# The digital edge: examining the relationship between digital competency and language learning outcomes

**DOI:** 10.3389/fpsyg.2023.1187909

**Published:** 2023-06-16

**Authors:** Jiafan Cao, G. Bhuvaneswari, Thangaraja Arumugam, B. R. Aravind

**Affiliations:** ^1^College of Education Science, Harbin Normal University, Harbin, China; ^2^School of Foreign Languages, Changchun Normal University, Chuangchun, China; ^3^School of Social Sciences and Languages (SSL), Vellore Institute of Technology, Chennai, India; ^4^Business School, Vellore Institute of Technology, Chennai, TN, India; ^5^School of Liberal Arts and Special Education (SLASE), Kalasalingam Academy of Research and Education, Krishnankoil, TN, India

**Keywords:** digital competency, didactic excellence, subjective excellence, technology literacy, knowledge deepening, educational methodologies, pedagogical excellence, educational technology

## Abstract

**Introduction:**

Technology in education, including language learning, is on the rise. Digital competency is essential for teachers to effectively integrate technology and enhance language teaching. It enables access to authentic materials, interactive exercises, and collaboration opportunities. However, integrating technology poses challenges for teachers.

**Objective:**

This empirical research aimed to investigate the impact of digital competency on language learning outcomes in the context of “smart education,” which incorporates sustainable practices and digital technologies in the language classroom.

**Methods:**

The study adopted a quantitative approach to collect and analyze data. The sample population for the study comprised of 344 language teachers at various language schools in a metropolitan city. The data collection carried out with a digital competency questionnaire. The data were analyzed using descriptive statistics and multivariate technique, i.e., structural equation modeling.

**Findings:**

The study found that digital competency positively correlated with language proficiency outcomes. Participants with higher levels of digital competency achieved better language learning outcomes compared to those with lower levels of digital competency. Additionally, the study found that incorporating sustainable practices, such as digitalized learning materials and virtual classrooms, positively contributed to language learning outcomes. The findings of this study suggest that digital competency plays a vital role in language learning outcomes in the context of “smart education.”

**Discussion & Recommendation:**

Teachers should consider incorporating digital tools and sustainable practices into their language teaching to enhance language learning outcomes. The study recommends that language educators should focus on developing digital competency and integrating sustainable practices into their language classroom to promote effective language learning.

## 1. Introduction

In recent years, the use of technology in education has become more and more common, and language learning is not an exception to this trend. The rapid growth of technology allows teachers to experiment with innovative methods with current digital natives. Presumably, the interaction hypothesis says that a language can be developed where there is regular interaction between the learners and other speakers, as technology continues to evolve and transform our daily lives, for successful integration of technology in language learning, it is crucial that language educators possess the required digital competencies.

According to Howard et al. (2021), digital competency encompasses the essential knowledge, skills, and attitudes necessary to utilize digital technologies in a responsible and effective manner. Digital proficiency could improve teaching and learning in language classrooms by giving students access to real-world language materials, interactive language exercises, and chances to interact and collaborate with native speakers and other students. Additionally, incorporating digital competency in language teaching can aid educators in offering a personalized and student-centered learning experience that caters to their students’ unique interests and requirements, as noted by Põldoja et al. (2014).

Integrating technology in the language classroom is more challenging. Teachers must navigate a vast array of digital tools and resources, adapt to the ever-changing technological landscape, and ensure that technology is used in an appropriate and effective manner (Mathai and Arumugam, 2016). At the same time, digital native or digital immigrant teachers must know about language learners to thrive in today’s digital age. In a Virtual Classroom, the live class is held on various video conferencing platforms, and online classroom apps and teachers and students engage in direct communication. As Basilotta-Gómez-Pablos et al. (2022) point out, lectures are often recorded in audio or video format and subsequently uploaded onto conferencing platforms or shared via email and other messaging platforms. Texts, reading materials, and assignments are shared via these platforms. A teacher needs to be digitally competent to perform all these tasks related to handling virtual classrooms (Thangaraja, 2016; Theobald and Bellhäuser, 2022).

This study aims to address the knowledge gap in the field of language teaching by investigating the role of digital competency in developing teachers’ performance (Chai et al., 2011). While there have been efforts to integrate digital technology in education, little research has been done on its impact on language teaching and learning, particularly from the perspective of language teachers. Therefore, the study seeks to explore the potential benefits and challenges of digital competency in language classrooms and its significance for teachers’ professional development.

This study focuses on the importance of digital competency in language classrooms, considering the critical role of classroom interaction in language learning and the desire to speak. To achieve this goal, the study will answer the following research questions: (i) How does digital competency improve the professional development of language teachers? and (ii) Are teachers who are digital immigrants or digital natives capable of supporting digital-native students in formal language classrooms? Hence, this paper includes the background of the study in literature review, methodology, and analyses of data and end with implications and conclusion.

## 2. Context of the study

### 2.1. Digital technology in education

Digital competence refers to a set of skills, knowledge, and practices that enable individuals to effectively utilize new technologies for various purposes, including learning, working, and leisure activities. In addition, it encompasses a proactive and responsible approach toward engaging with the digital world (Ahmed et al., 2022). It has been asserted that societal technology innovation is dramatically altering the landscape of education (Zainal and Zainuddin, 2020). Language learning techniques may also be affected by the learner’s objectives, the sense of the learning situation, and the learner’s society’s cultural values (El Said, 2015). Differentiated applications of more complex behaviors, such as participating in interactive digital media aspects, may be common (Goodhue and Thompson, 1995).

The definition of the teacher as a facilitator underscores the significance of the learner’s active participation in the learning process. In this approach, the teacher’s role is to support and guide the student in their learning journey by creating a suitable learning environment and providing necessary resources (Edelmann et al., 2023). Defining Digital competency requires an implied approach for realistic educational applications: digital technology should be integrated into both learning and teaching in a pervasive manner. Regardless of the capabilities of the digital medium or the mode of instruction, a teacher must develop a student’s skills, increase their ability to learn, and gain knowledge, and this is a universally agreed principle of Lozano Rivas et al. (2023).

Teaching patterns, criteria, and standards have changed in a variety of ways, including delivery models, students’ needs, and technology (DeVries et al., 2020). Preparing and maintaining the requisite skills required to teach in today’s changing world takes a greater commitment than ever before (Al Khateeb, 2017). Even though the teachers are reluctant about how the students would use the technology, teachers who used it in their own lives felt more comfortable transitioning that experience into their classrooms (Bucur and Popa, 2017). Teachers who are naturally oriented toward constructivist teaching are more likely to use technology to inspire student experimentation and learning (Mehrvarz et al., 2021). The constant shift in technological advances is a defining feature of this technological age. Technical sources and implementations are constantly being improved (Silva and Behar, 2019). The practical application of digital technology as a learning tool and the development of digital technology skills within programs that go beyond the conventional practice of restricting LMS, and email use are highly desired (Basilotta-Gómez-Pablos et al., 2022; Fernández-Sánchez et al., 2022). According to Benali et al. (2018), educational technology applications have the potential to cultivate various skills beyond test preparation, including knowledge literacy, communication skills, global awareness, imagination, and teamwork.

### 2.2. Digital competency

Technology is profoundly influencing our communication processes, everyday experiences, and, as educators, our instructional resources and learning environments (Erskine et al., 2019). Digital identity should be consciously taught in pre-service instructor courses in higher education, and students and scholars should accept the technologies associated with social and digital media. There is a particular need for phenomenological and ethnographic studies that look at how digital texts can either challenge or reinforce classroom culture (Shahzad et al., 2021).

Over the last 10 years, there have been significant advancements in online learning. Faculty are now realizing how much time it takes to develop and deliver a high-quality online course, and students are realizing how difficult it is to complete courses and degree programs online (Mohammadyari and Singh, 2015). By utilizing a variety of technology-mediated resources and applications, teachers can enhance language learning while also creating transferable digital literacies (Curran-Everett, 2019). VLEs, also known as electronic course management tools, are online spaces that allow teachers to electronically coordinate their own and their students’ work (Akkuzu, 2014). Based on previous evidence, the hypothesis has been framed as follows:

*H1*: Digital competency has an impact on the teacher’s professional development.

## 3. Components of digital competency

### 3.1. Technology literacy

The TTF model posits that individuals’ inclination toward assessing and comprehending technical capabilities can facilitate their job performance. This theory suggests that users’ abilities and skills can help technology align with their task requirements, resulting in improved performance. To achieve this, individuals require a set of competencies, known as digital competency (DC), which encompasses their values, responsiveness, interaction, and innovation in using digital tools and participating in the digital environment (Ezziane, 2007). DC is necessary for individuals to carry out work functions, problem-solving, communication, information management, collaboration, document creation and sharing, and knowledge development (Ezziane, 2007).

The concept of technology competence, also known as digital competency, has been recognized as a crucial phenomenon in the field of Information Systems (IS), as noted by Omotayo and Haliru. Digital Competency (DC) is a comprehensive concept that has been included as a separate element in the Technology Acceptance Model (TTF) theory and has demonstrated its usefulness in a variety of information systems research contexts. In our study, DC is composed of four distinct elements, namely technology literacy, knowledge depth, presentation skills, and professional abilities, which have been identified by Szabo et al. (2002).

A fundamental technological skill that is referred to as technology literacy is the ability to use and handle any technology or tool (TL). The TTF model asserts that when humans can use technology, it will be appropriate for both the duties it endorses. This implies that if users’ abilities and technology capabilities are compatible, they can use technology to complete their tasks (Hasse, 2017). Users’ technical literacy, also known as digital literacy, plays a significant role in utilizing instructional resources (Mohammadyari and Singh, 2015). Therefore, it is important to consider this aspect when evaluating the impact of materials on task performance.

### 3.2. Knowledge deepening

Knowledge deepening (KD) refers to teachers’ mastery of ICT tasks, such as critical thinking, problem-solving, data analysis, policy and information management, and skill application, KD is an important component of instructors’ IS expertise (Weiss and Miller, 2006).

The development of knowledge-deepening abilities through digital literacy is another part of digital competency in language classrooms. Digital literacy is the capacity to effectively and morally access, assess, and use digital information (Sanjeev et al., 2022). Digital literacy is crucial for both teachers and students in the language classroom. Students must be able to use digital resources to access, evaluate, and share information in the target language, and teachers must be able to use these tools to design and deliver language learning materials (Fan, 2022). According to research, acquiring digital literacy abilities can help kids become more fluent language users and expand their capacity for critical thought (Kirschner and De Bruyckere, 2017).

### 3.3. Presentation skills

In contrast, educators who employ didactic instruction, also known as presentation skills (PRS), communicate with their students by providing detailed explanations, descriptions, lectures, posing questions, addressing their queries, and building a rapport with them. According to Curran-Everett (2019), enhanced presentation skills allow individuals to engage with their activities and connect with others in any setting. A recent mixed-method study found that users’ presentation skills can help overcome barriers to technology usage, leading to improved task performance (Serrano and Karahanna, 2016).

### 3.4. Professional skills

Professional skills (PFS) is another crucial component of DC that is essential for teaching staff to succeed in their job. In addition to attending courses, programs, conferences, seminars, events, and workshops, educators acquire their knowledge and expertise through experimentation, observation, professional networks, and personal connections, as highlighted by Unesco (2011). According to Akkuzu (2014), individuals who have confidence in their prior ICT skills or are comfortable using previous technology are more proficient in utilizing new materials compared to novices. A group of academics believes that acquired knowledge is related to creative work. The work can be completed since academic research has demonstrated a substantial correlation between prior technological experience and human-technology interaction (Abykanova et al., 2016).

The significance of digital technologies in language teaching and learning has increased in recent years. The importance of digital literacy in language instruction has been extensively discussed in various literature. This literature review provides an overview of prior research on the topic, and the following hypotheses are proposed:

*H2*: Technology literacy has an impact on digital competency.

*H3*: Knowledge deepening has an impact on digital competency.

*H4*: Presentation skills have an impact on digital competency.

*H5*: Professional skills have an impact on digital competency.

### 3.5. Teacher’s professional development

The integration of digital technologies to enhance language learning is an essential aspect of digital literacy in the language classroom. It enhances the teacher’s effectiveness by enabling them to leverage mobile devices like smartphones and tablets to access online resources, participate in virtual language exchanges, and complete language learning assignments. Studies indicate that the use of such tools can significantly enhance students’ motivation, autonomy, and engagement in language learning (Warschauer and Matuchniak, 2010).

Teachers’ professional development can also help with Pedagogical excellence, Subject and didactic excellence through digital collaboration and intercultural communication (Liu et al., 2021). With the aid of digital tools, students can interact with classmates from other backgrounds, share ideas and viewpoints, and take part in group language-learning activities. According to research, these activities can improve students’ communication and intercultural competency and it improves teachers’ professionalism (Belz and Thorne, 2006).

Yet, using digital technologies in language classrooms is not without its difficulties. One difficulty is that teachers must possess the technical know-how and abilities to successfully incorporate digital tools into their instruction. Making ensuring that digital tools are used morally and in a way that encourages equal access to opportunities for language acquisition for all pupils is another difficulty (Warschauer and Matuchniak, 2010). It increases the pedagogical excellence in their language classrooms.

Overall, the research indicates that digital proficiency is crucial in language instruction. The use of digital technologies can improve language acquisition, foster the development of digital literacy skills, and promote cross-cultural cooperation. It increases the excellence of didactic and subject in their language classrooms. To effectively include digital technologies in education, however, teachers must possess the requisite technical expertise and experience, and when employing these tools, ethical considerations must be considered. From the past evidence, the hypotheses were formulated:

*H6*: Teachers’ professional development has an impact on Pedagogical excellence.

*H7*: Teachers’ professional development has an impact on Didactic excellence.

*H8*: Teachers’ professional development has an impact on Subject excellence.

## 4. Method and analysis

**Figure 1:**
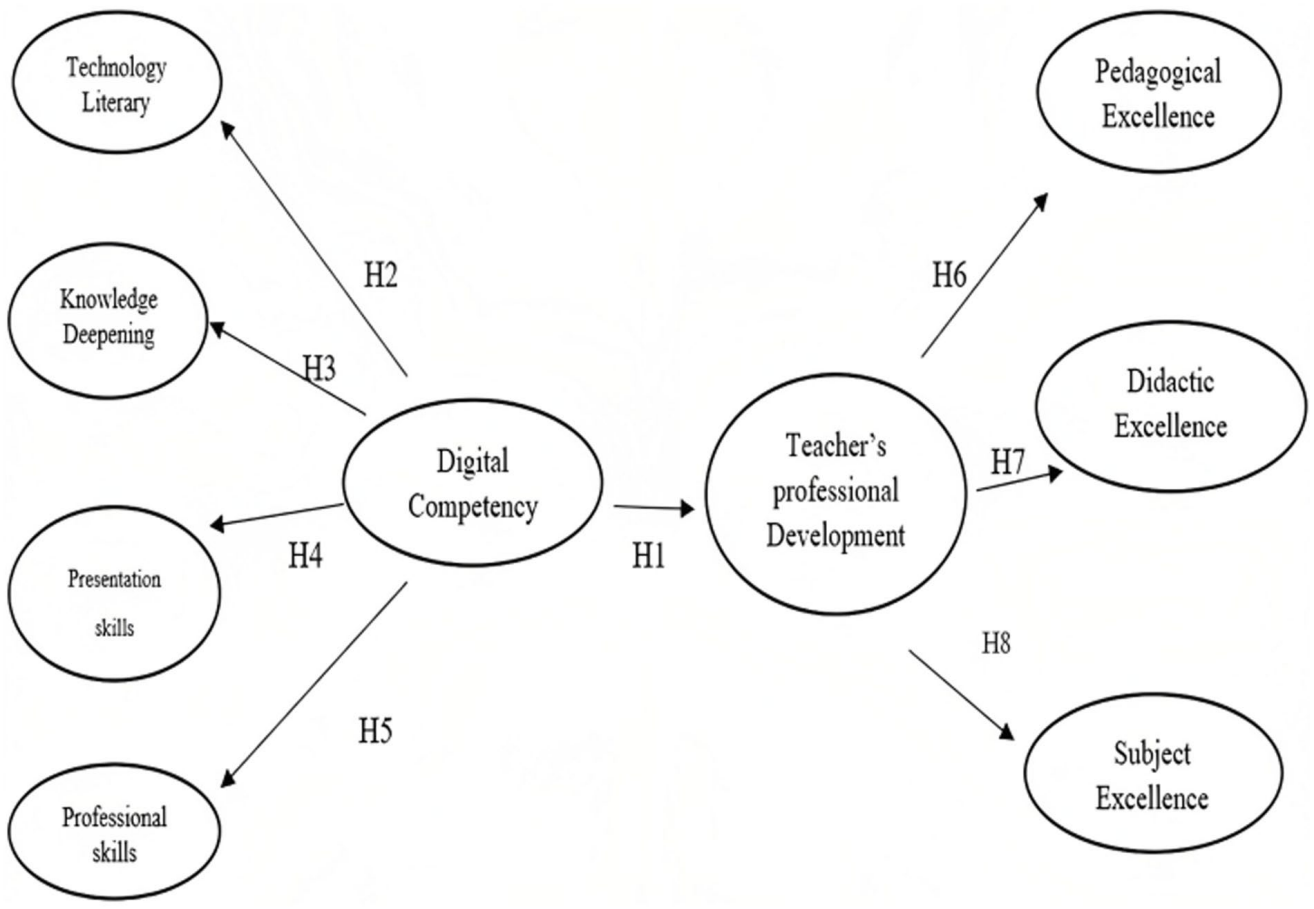
Proposed conceptual model.

The methodology employed in this study was quantitative and aligned with the positivist paradigm. A deductive approach was used, along with a cross-sectional design and a survey strategy. The positivist paradigm aims to establish causal relationships that demonstrate how independent variables influence dependent variables. To test the hypotheses, a deductive approach was adopted, building on existing research and developing a conceptual model (Figure 1). The study was essentially cross-sectional since data was collected only once at a specific point in time.

We created an online survey through convenience sampling, targeting academic professionals, including language teaching staff. However, data collection was challenging due to issues with the sample frame, which made it difficult to gather responses from the intended participants. Because of this, convenience sampling turned out to be the most efficient method of choosing potential study participants. To contact the respondents and discuss the goals and parameters of the study, we spoke with the main contacts of several educational institutions. All participants were persuaded to respond to an online invitation to complete the survey. The survey lasted nearly eight weeks. Finally, 410 people took the survey anonymously. However, only 344 valid responses were received.

In the questionnaire section of this study, utilized two categories: one category targeted variables associated with the primary research, while the other collected demographic variables, such as age, gender, and educational background. The study utilized 12 items adapted from previous work to measure the four key aspects of digital competency, namely technology literacy (three items), knowledge deepening (three items), presentation skills (three items), and professional skills (three items; Hizam et al., 2021). These items were rooted in existing literature and were assessed on a five-point Likert scale ranging from 1 (strongly disagree) to 5 (strongly agree). Similarly, to assess three aspects of a teacher’s professional development which includes pedagogical excellence (10 items), didactic excellence (six items), and subject excellence (six items) adopted measurement scale from study of Makovec (2018). These items were also rated on a five-point Likert scale ranging from 1 (strongly disagree) to 5 (strongly agree), adhering to the predetermined guidelines outlined in the previous researchers’ works. To analyze the data, descriptive analysis and PLS-SEM were used. Because descriptive analysis provides concise summary of demographic data and PLS-SEM provides statistical measures, such as path coefficients and *p* values, to determine the strength and significance of these relationships.

### 4.1. Statistical analysis

The likelihood that categorical data will occur (percentages) and the averages, as well as standard deviations (SD) of the dependent variable, were all summarized using descriptive statistics.

Each gender category within the total sample. For instance, males account for 45.1% (155 out of 344) of the population, while females make up 54.1% (186 out of 344). The percentage of individuals in each age category within the total sample. For example, individuals below the age of 30 account for 27.3% (94 out of 344) of the population. Similarly, individuals aged between 30 and 40 represent 25.6% (88 out of 344), those aged between 41 and 50 make up 24.4% (84 out of 344), and individuals above the age of 50 comprise 22.7% (78 out of 344). The percentage of individuals with each educational qualification within the total sample. For example, individuals with a postgraduate qualification account for 39.2% (135 out of 344) of the population. Similarly, individuals with a Master of Philosophy degree represent 34.0% (117 out of 344), those with an unspecified “3” qualification make up 0.9% (3 out of 344), and individuals with a Doctor of Philosophy degree comprise 25.9% (89 out of 344).

Structural equation modeling is the finest statistical method for assessing *a priori* models, finding results, and identifying directed indirect relationships between variables (SEM). Partial Least Squares Structural Equation Modeling (PLS-SEM) and Covariance-based Structural Equation Modeling (CB-SEM) are two approaches used in structural equation modeling. While both methods are used to analyze complex relationships between latent variables, they differ in their underlying principles and assumptions. This study used PLS-SEM, because it is more suitable for exploratory research or when the emphasis is on prediction. This study investigated the predicted connections between digital competence and teachers’ professional development. The associations among exposure, result, and path coefficient were examined using analysis.

### 4.2. Reliability and validity test

In evaluating the effectiveness of the measurement approach, it is crucial to consider the construct validity and reliability of the study’s constructs. We used Cronbach’s alpha (CA) and composite reliability (CR) to assess the reliability of the constructs. Cronbach’s alpha test produces results between 0 and 1, with a score above 0.7 indicating strong internal consistency, while a value between 0.5 and 0.7 suggests moderate consistency (Hair et al., 2010; Hinton et al., 2014). All the Cronbach’s alpha values in Table 1 were above 0.5, indicating that the tested items’ outcomes are sufficiently reliable to support this research. Researchers, Kock and Lynn (2012) assert that when evaluating construct validity, Cronbach’s alpha and composite reliability are useful tools. All constructs, including didactic excellence (CR = 0.837, CA = 0.823), digital competency (CA = 0.871, CR = 0.905), knowledge deepening (CA = 0.752, CR = 0.758), Pedagogical excellence (CA = 0.881, CR = 0.893), presentation skills (CA = 0.753, CR = 755), professional skills (CA = 0.846, CR = 852), subjective excellence (CA = 0.869, CR = 873), Teacher’s professional development (CA = 0.935, CR = 0.94), and Technology literacy (CA = 0.32, CR = 0.72,), are below the acceptable level, as shown in Table 2. Although the technology literacy levels are below 0.7, they are not excessively low and are still considered to be reliable indicators for the conclusions.

**Table 1:** Convergent validity.

Constructs	Average variance extracted (Ave)
Didactic excellence	0.54
Digital competency	0.46
Technology literacy	0.53
Pedagogical excellence	0.49
Presentation skill	0.67
Professional skill	0.76
Subjective excellence	0.61
Teachers’ professional development	0.42

**Table 2:** Constructs reliability and validity.

Constructs	Cronbach’s alpha (Ca)	Composite reliability (Rho_A; Cr)
Didactic excellence	0.82	0.84
Digital competency	0.87	0.90
Technology literacy	0.32	0.72
Pedagogical excellence	0.88	0.89
Presentation skill	0.75	0.75
Professional skill	0.85	0.85
Subjective excellence	0.87	0.87
Teachers’ professional development	0.93	0.94

### 4.3. Convergent validity

The study employed factor analysis to examine convergent validity, which assesses the reliability of questionnaire responses to support the research findings (Fornell and Larcker, 1981). The questionnaire comprised 30 Likert scale questions across five components. The Average Variance Extracted (AVE) was used as a crucial index for analyzing convergent validity in Confirmatory Factor Analysis (CFA). To establish convergent validity, each item loading must be at least 0.5 and statistically significant, indicating a correlated value of *p* equal to or less than 0.05. Moreover, each latent construct’s AVE should be at least 0.5, following criteria set by Hair et al. (2010), Kock and Lynn (2012), and Fornell and Larcker (1981) to ensure construct validity. Table 1 displays the AVE values for the nine factors, where values greater than 0.5 indicate higher effectiveness (Dos Santos and Cirillo, 2021). Even though the AVE value of 0.5 has not yet been reached by pedagogical excellence and teachers professional development, the figures are not excessively low and are nevertheless considered to be reliable indicators of the findings.

### 4.4. Discriminant validity—Fornell-Larcker criterion

The Heterotrait-monotrait (HTMT) ratio was used to support the discriminant validity of the Fornell-Larcker criterion. For diagonal values (AVEs) of Table 3 to be discriminant, they must have larger square roots than the off-diagonal coefficients (Fornell and Larcker, 1981; Hair et al., 2010; Kock and Lynn, 2012). The findings show that three-factor loadings have discriminant validity.

**Table 3:** Discriminant validity—Fornell-Larcker criterion.

Constructs	DE	DC	KD	PE	PRS	PFS	SE	TPD	TL
Didactic excellence	0.73								
Digital competency	0.73	0.68							
Knowledge deepening	0.75	0.86	0.82						
Pedagogical excellence	0.67	0.73	0.67	0.70					
Presentation skill	0.61	0.89	0.67	0.63	0.82				
Professional skill	0.44	0.78	0.48	0.46	0.67	0.87			
Subjective excellence	0.71	0.80	0.71	0.73	0.66	0.55	0.78		
Teachers’ professional development	0.86	0.84	0.79	0.92	0.71	0.54	0.90	0.65	
Technology literacy	0.68	0.83	0.73	0.71	0.64	0.46	0.80	0.81	0.73

### 4.5. HTMT: discriminant validity

To examine the discriminant validity of the study’s latent constructs, we utilized HTMT ratios. HTMT ratios should be less than 0.85 for ideal performance (Henseler et al., 2015). Additionally, Sandström et al. (2005) have stated that HTMT ratios must be below 0.90. As displayed in Table 4, all constructs have discriminant validity.

**Table 4:** HTMT—discriminant validity.

Constructs	DE	DC	KD	PE	PRS	PFS	SE	TPD	TL
Didactic excellence									
Digital competency	0.85								
Knowledge deepening	0.95	1.04							
Pedagogical excellence	0.77	0.85	0.80						
Presentation skill	0.76	1.08	0.88	0.77					
Professional skill	0.52	0.9	0.57	0.52	0.84				
Subjective excellence	0.82	0.89	0.86	0.82	0.81	0.63			
Teachers’ professional development	0.97	0.91	0.92	1.01	0.84	0.59	0.99		
Technology literacy	0.99	1.19	1.17	0.97	0.96	0.61	1.08	1.08	

### 4.6. Structural equation modeling

**Figure 2:**
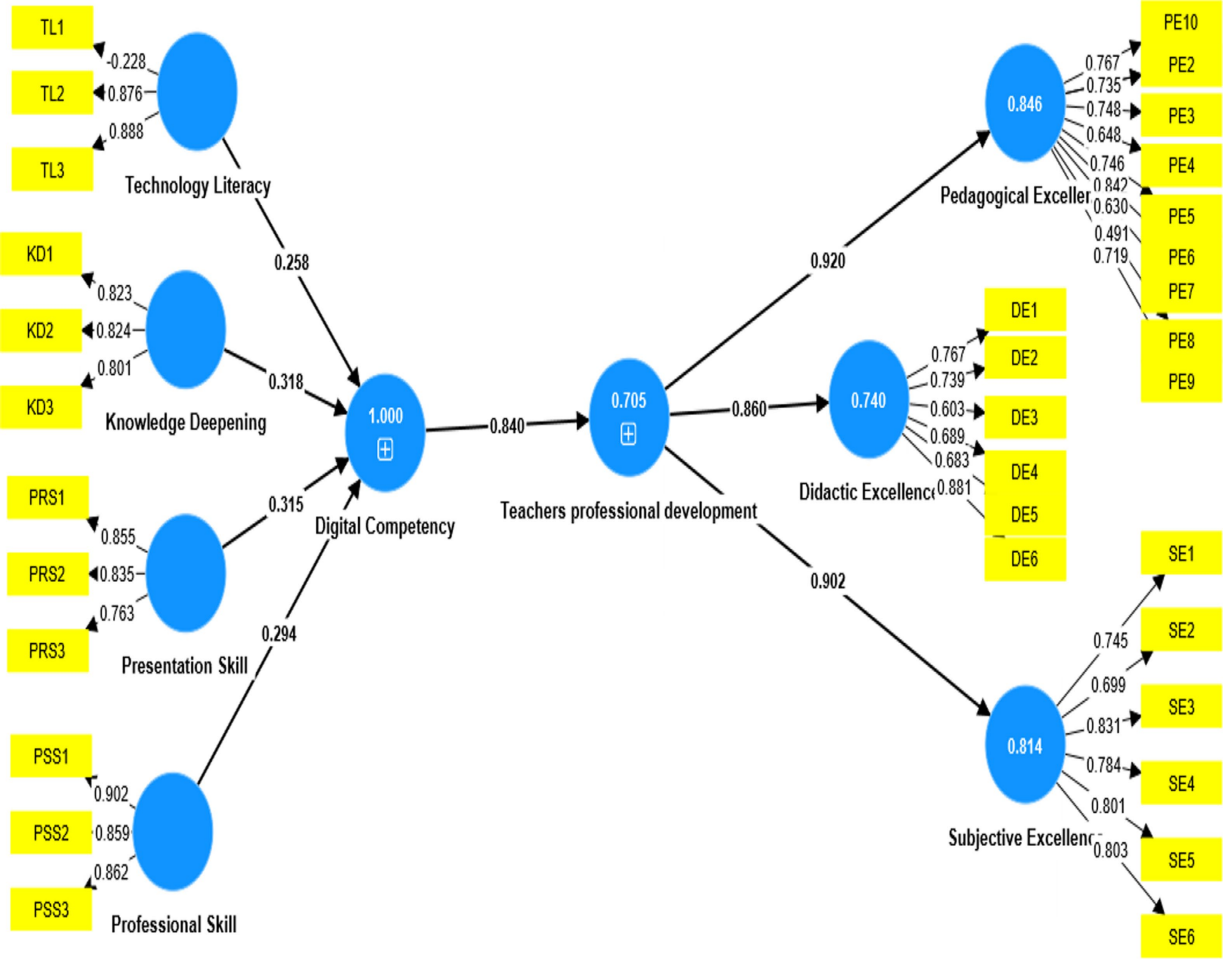
Structural equation modeling.

The research framework shown in Figure 2 was examined by using the structural equation modeling (SEM) technique, which assesses the psychometric properties of measurement models. It also evaluates the structural model’s variables. Smart PLS creates a component-based solution to structural equation modeling by using the bootstrapping technique. The Smart PLS route design also needs two parts: an outside model (a structural equation) as well as an interior framework. The working research model’s hypotheses for determining the correlation between IV and DV as well as any intervening factors are listed below. These theories are supported by a range of RF intensities. In addition to these hypotheses, several working hypotheses examine how the different dimensions of the primary constructs relate to those of the intermediate constructs.

With path coefficients of =0.840, respectively, Figure 2 illustrates the statistically significant relationship between digital competency and teachers’ professional development. The results show that if the language teacher has high digital competency, it would increase the teacher’s professional development in their job. H1 is therefore supported.

Additionally, the results demonstrate that the four components with a digital competency purpose have a statistically significant impact with a path coefficient above 0.25, which is technology literacy (0.258), knowledge deepening (0.318), presentation skills (0.315), and professional skills (0.294). The results show that digital competency will improve when language teachers have the ability of knowledge deepening, technology literacy, professional skills, and presentation skills. As a result, H2, H3, H4, and H5 are supported. The findings show that the three components of a teacher’s professional development purpose, pedagogical excellence (0.920), Didactic excellence (0.860), and subjective excellence, have a statistically significant impact with a path coefficient greater than 0.80 (0.906).

The results show that teachers’ professional development will increase the excellence of pedagogy, subject and didactic among teachers in language classrooms. H6, H7, and H8 are therefore supported. The statistical significance and positivity of each path coefficient (β) are both high (*p* < 0.05). the result of path coefficients has been shown in Table 5.

**Table 5:** Path coefficient.

Constructs relationship	Path coefficients
Digital competency ➝Teachers’ professional development	0.84
Knowledge deepening ➝Digital competency	0.32
Presentation skill ➝Digital competency	0.32
Professional skill ➝Digital competency	0.29
Teachers’ professional development ➝Didactic excellence	0.86
Teachers’ professional development ➝Pedagogical excellence	0.92
Teachers’ professional development ➝Subjective excellence	0.90
Technology literacy ➝Digital competency	0.26

## 5. Discussion

One of the most important qualities that teachers need to possess in today’s culture is digital competence, which has gained significant relevance in the educational context. While many models and frameworks concentrate on preparing students for university education, there is an increasing emphasis on evaluating the digital competencies of university teachers. Digital competencies encompass a wide range of knowledge, abilities, and attitudes that educators must possess to proficiently leverage technology in their teaching methodologies (Basilotta-Gómez-Pablos et al., 2022). A recent study of Kennedy-Clark and Reimann (2022) emphasized the important role that technology plays in the knowledge systems of teachers. The study also found that, despite having access to resources, universities still have room for improvement in their digital competencies at an institutional level. We find that most of the research in the study examines how teachers enhance their digital competencies through professional development. The statistical data shows a low to medium-low level of digital competence among teachers, particularly in the use of 2.0 tools for assessment and addressing issues using digital technology. Teachers are aware of these shortcomings, especially in evaluating educational practices, and are requesting pedagogical support to integrate technology into all subject areas instead of isolating it. Similar results have been reported in other studies. For instance, Demeshkant (2020) found that academic teachers in Poland had a moderate level of digital competency in both pedagogical and technological knowledge. Additionally, Santos et al. (2021) observed that higher education teachers in Portugal had a low level of digital competency in assessment, according to the DigCompEdu model.

Despite the awareness among teachers that the use of digital technology and data literacy can enhance decision-making, many struggle with using conventional teaching methods, which they may dislike (Agustini et al., 2019). Consequently, there is a need for a comprehensive framework for teachers’ professional development that incorporates digital competence. This study emphasizes the importance of integrating teachers’ traditional technological, pedagogical, and subject-matter knowledge with their digital competence. The study’s results revealed correlations between various aspects of the Digital Competency framework, including technology literacy, knowledge deepening, presentation skills, and professional skills, and teaching professional development, such as pedagogical excellence, Didactic excellence, and subject excellence. These findings support the notion that technology literacy and knowledge deepening, as well as presentation skills and professional skills, are crucial components of teachers’ core professional development (Revuelta-Domínguez et al., 2022). The implication of these findings is that to facilitate data-driven decision-making in education, smart learning environments must integrate technology literacy, knowledge expansion, and professional skills (Chai et al., 2011). This paradigm raised awareness of the link between teachers’ digital proficiency and improved professional development, data literacy, and cross-disciplinary pedagogical excellence.

The findings showed that the overall influence of digital competency on educators’ professional development is strong, with a path coefficient of 84% and a regression change of 70% for teachers’ professional growth. These findings, which concur with those of the study of Pera et al. (2022) which also identified differences and gaps in professors’ and teachers’ digital knowledge and abilities, may have important policy ramifications for those in the policy-making and educational communities who are dedicated to ensuring the quality of education. The results of the study of Tai et al. (2022) showed that teacher-driven professional development programmers can enable instructors to take control of their students’ learning experiences because they acknowledge teachers as professionals with important insights for teachers’ ongoing progress. According to the study of Cui and Zhang (2022), many teachers were competent practitioners with some room for development.

### 5.1. Theoretical and practical implications

Integration of technology in language education: The study emphasizes the significance of digital competency in language classrooms and its impact on teachers’ professional development. This finding contributes to the existing literature by highlighting the role of technology in language education and the need for language teachers to possess digital competencies to effectively utilize technology in their teaching practices. This research contributes to the theoretical understanding of how these techniques can be used to evaluate the effectiveness and distinctiveness of digital competency in language education research. These insights contribute to the broader theoretical understanding of language teaching and learning in the digital age.

Language teachers should focus on improving their digital competencies to support their professional development and effectively integrate technology into language classrooms. Teachers should pay attention to components such as knowledge deepening, presentation skills, professional skills, and technology literacy to enhance their digital competency and improve language teaching practices. Teachers’ professional development should encompass not only digital competency but also pedagogical excellence, didactic excellence, and subjective excellence. Addressing all these aspects can contribute to overall growth as language educators. Schools and educational institutions should provide targeted training programs to support teachers in developing their digital competencies and understanding how to effectively utilize technology in language classrooms. Regular assessments of constructs’ reliability and validity should be conducted to ensure the accuracy and consistency of the measurement approach. Areas that fall below the desired thresholds should be identified and addressed for continuous improvement. Finally, the study highlights the importance of digital competency and its relationship to teachers’ professional development in language classrooms. By addressing the identified implications, language educators can better navigate the digital landscape and enhance their teaching practices for improved student outcomes.

## 6. Conclusion

The study yielded numerous benefits for the participants, including teachers, students, and professors. By encouraging diverse stakeholders to contribute to a specific framework, the study facilitated a more holistic approach to teaching that takes various perspectives into account. The development of digital competency promotes learning by enabling individuals to derive meaning from their actions through observing and engaging with others, as evidenced by the “looking-glass self” concept (Stevanovic and Weiste, 2017). This topic encouraged educators to deepen their understanding of technology’s role in the classroom, ultimately resulting in the improvement and refinement of digital competency (Gkevrou and Stamovlasis, 2022). While the teachers acknowledged that they had not met all requirements, their integrated model of teachers’ data literacy offered a roadmap for future improvement. This research has significant implications for student learning, educational policies, curricula, institutions, and long-term teaching provision (Liu and Zhang, 2021). The research methodology employed in this study facilitated the integration of teachers’ knowledge and understanding of digital competency, uncovering critical factors, cognitive processes, and a consensus perspective. This conceptual framework for knowledge- or skill-based learning has the potential to drive curricular reforms guided by teachers (Henderson and Corry, 2021). To improve their teaching practices, it is important for teachers to take advantage of opportunities to enhance their didactic and subject excellence in technology and data applications (Chatzopoulos et al., 2023). They should also have a positive mindset toward integrating technology, experience, and pedagogical content knowledge (Rohaan et al., 2012).

## Data availability statement

The original contributions presented in the study are included in the article/supplementary material, further inquiries can be directed to the corresponding author.

## Author contributions

GB: conceptualization. TA: methodology and software. TA and GB: validation. BA: formal analysis, data curation, visualization, and project administration. JC: investigation, resources, supervision, and funding acquisition. GB and JC: writing—original draft preparation. GB and TA: writing—review and editing. All authors contributed to the article and approved the submitted version.

## Funding

This project was funded by Construction of Comprehensive Quality Evaluation System of Postgraduates from the Perspective of All-round Talent (JJKH20230884SK) and The Reform of English Major Curriculum System in Local Normal Colleges under the Background of Key Competence (JGJX2019D231).

## Conflict of interest

The authors declare that the research was conducted in the absence of any commercial or financial relationships that could be construed as a potential conflict of interest.

## Publisher’s note

All claims expressed in this article are solely those of the authors and do not necessarily represent those of their affiliated organizations, or those of the publisher, the editors and the reviewers. Any product that may be evaluated in this article, or claim that may be made by its manufacturer, is not guaranteed or endorsed by the publisher.

## Supplementary material

The Supplementary material for this article can be found online at: https://www.frontiersin.org/articles/10.3389/fpsyg.2023.1187909/full#supplementary-material
