# Supplementary material for: The digital edge: examining the relationship between digital competency and language learning outcomes
Source: Front Psychol. 2023 Jun 16;14:1187909. doi: 10.3389/fpsyg.2023.1187909 (PMC10313065; doi:10.3389/fpsyg.2023.1187909)
Supplement: Supplementary file 1 [file Data_Sheet_1.docx]

**QUESTIONNAIRE ON**

**THE DIGITAL EDGE: EXAMINING THE RELATIONSHIP BETWEEN DIGITAL COMPETENCY AND LANGUAGE LEARNING OUTCOMES**

To be filled by Teachers/ Professors

The purpose of the study in general and this questionnaire in particular is to study on “The Digital Edge: Examining the Relationship Between Digital Competency and Language Learning Outcomes” which may help educators to understand their digital competency and learning outcome of students. The research is undertaken as academic requirements of Ph.D. It also helps to gain practical knowledge on the topic under investigation and other prospective researchers as a stepping stone to carry out further investigation. I want to assure that this research is only for academic purpose. Your cooperation will be highly appreciated in this regard.

General Instructions

In all cases where answer options are available, please put tick (✅) in the appropriate box. This Questionnaire is divided in to three sections

*Section A: Demographic information*

*Section B: Digital competency and Techer’s professional development*

**Section A**

**Demographic Details**

Age

- Below 30
- 3-40
- 41-50
- Above 50

Gender

- Male
- Female

Educational background

- PG
- M Phil
- Ph.D.

**Section B**

***Digital competency and Techer’s professional development***

Questions are related to understanding digital competency on learning outcome. Listed below are statements about Teacher professional development and digital competency.

Please put tick (✅)

| Items | Strongly Disagree | Disagree | Neutral | Agree | Strongly Agree |
| --- | --- | --- | --- | --- | --- |
| **Digital competency** | | | | | |
| **Technology Literacy** | | | | | |
| I could adapt my searches based on knowledge about how search engines produce results |  |  |  |  |  |
| I could store and organize digital content in a way so I can later find it |  |  |  |  |  |
| I could use search engines to find a given type of information, e.g. images, videos or maps |  |  |  |  |  |
| **Knowledge deepening** | | | | | |
| I could learn a new programming language on my own |  |  |  |  |  |
| I could adapt my ways of working based on new digital tools |  |  |  |  |  |
| I could construct a product with the support of digital technology |  |  |  |  |  |
| **Presentation skills** | | | | | |
| I could correctly cite the creator when using or disseminating another people’s material |  |  |  |  |  |
| I could combine tools in order to create digital content |  |  |  |  |  |
| I could summarize information from different sources in a representative manner |  |  |  |  |  |
| **Professional skill** | | | | | |
| I could recognize hate speech in discussions online |  |  |  |  |  |
| I could arrange an online meeting as a replacement for a physical meeting |  |  |  |  |  |
| I could choose a suitable creative commons license for material I have created |  |  |  |  |  |
| **Teacher’s Professional Development** | | | | | |
| **Pedagogical Excellence** | | | | | |
| My students take part in creating classroom rules |  |  |  |  |  |
| I make decisions on solving disciplinary offences together with the students. |  |  |  |  |  |
| I talk to the students whom I assume have personal problems. |  |  |  |  |  |
| I take responsibility for solving educational and professional problems |  |  |  |  |  |
| My rules about discipline and behaviour in the classroom are clear |  |  |  |  |  |
| I am consistent. |  |  |  |  |  |
| I am decisive. |  |  |  |  |  |
| When planning a lesson, I consider the educational effect that the content could achieve. |  |  |  |  |  |
| I encourage my students’ sensitivity for art and artistic expression. |  |  |  |  |  |
| I emphasize the value dimension of the learning content. |  |  |  |  |  |
| **Didactic Excellence** | | | | | |
| I plan lessons in detail |  |  |  |  |  |
| I structure lessons clearly following the established sequence of didactic phases (e.g. introduction, Taking account of presentation, practice, revision, testing, assessment). |  |  |  |  |  |
| I prepare lesson plans for each individual lesson. |  |  |  |  |  |
| I define operational learning goals for each lesson. |  |  |  |  |  |
| I adapt lessons to my students’ individual each class’s specific characteristics (their interests, prior knowledge, characteristics learning styles). |  |  |  |  |  |
| When planning work in the classroom, I take account of the characteristics of the class I teach. |  |  |  |  |  |
| **Subjective Excellence** | | | | | |
| I participate in continuing education and training. |  |  |  |  |  |
| I read professional literature. |  |  |  |  |  |
| I include new developments meaningfully in my teaching. |  |  |  |  |  |
| I follow development in my subject of expertise |  |  |  |  |  |
| I am fully acquainted with the legal and other formal rules that affect my pedagogical work. |  |  |  |  |  |
| In my professional pedagogical work, I consistently follow the formal framework of rules defined by legislative and other documents |  |  |  |  |  |
